# Sentinel surveillance of child maltreatment cases presenting to Canadian emergency departments

**DOI:** 10.1186/s12887-019-1788-9

**Published:** 2019-10-29

**Authors:** Aimée Campeau, Lil Tonmyr, Erik Gulbransen, Martine Hébert, Steven McFaull, Robin Skinner

**Affiliations:** 1 Public Health Agency of Canada, Ottawa, ON Canada; 2 Independent Researcher, Burnaby, BC Canada; 3 Université du Québec à Montréal, Montreal, QC, Canada

**Keywords:** Child maltreatment, Physical abuse, Sexual abuse, Neglect, Exposure to family violence, Emergency department data, Public health surveillance

## Abstract

**Background:**

The Canadian Hospitals Injury Reporting Prevention Program (CHIRPP) is a sentinel surveillance program that collects and analyzes data on injuries and poisonings of people presenting to emergency departments (EDs) at 11 pediatric and eight general hospitals (currently) across Canada. To date, CHIRPP is an understudied source of child maltreatment (CM) surveillance data. This study: (1) describes CM cases identified in the CHIRPP database between1997/98 to 2010/11; (2) assesses the level of CM case capture over the 14-year period and; (3) uses content analysis to identify additional information captured in text fields.

**Methods:**

We reviewed cases of children under 16 whose injuries were reported as resulting from CM from 1997/98 to 2010/11. A time trend analysis of cases to assess capture was conducted and content analysis was applied to develop a codebook to assess information from text fields in CHIRPP. The frequency of types of CM and other variables identified from text fields were calculated. Finally, the frequency of types of CM were presented by age and gender.

**Results:**

A total of 2200 CM cases were identified. There was a significant decrease in the capture of CM cases between 1999 and 2005. Physical abuse was the most prevalent type (57%), followed by sexual assault (31%), unspecified maltreatment (7%), injury as the result of exposure to family violence (3%) and neglect (2%). Text fields provided additional information including perpetrator characteristics, the use of drugs and/or alcohol during the injury event, information regarding the involvement of non-health care professionals, whether maltreatment occurred during a visitation period with a parent and, whether the child was removed from their home.

**Conclusions:**

The findings from this initial study indicate that CHIRPP could be a complimentary source of CM data. As an injury surveillance system, physical abuse and sexual assault were better captured than other types of CM. Text field data provided unique information on a number of additional details surrounding the injury event, including risk factors.

## Background

Child maltreatment (CM) includes physical and sexual abuse, neglect, emotional maltreatment, and exposure to intimate partner violence (IPV) [1]. It has been well established that CM adversely impacts the development and well-being of children throughout their lives [2]. To understand the scope of this problem, evidence is collected from multiple sources such as child welfare data, police data, mortality and morbidity data, and population health and social surveys [3]. Examining information from diverse data sources can help to provide a more nuanced picture of CM than can be gained from any one source [4–6]. For instance, the Canadian Incidence Study of Reported Child Abuse and Neglect (CIS) provides information regarding reported CM investigations by child welfare [7]. However, official substantiated cases of CM only represent the “tip of the iceberg” as they do not include information about unreported CM or cases investigated by the police [7]. Self-reported population health data provides the most accurate national estimates of CM, although concerns have been raised regarding the willingness of respondents to disclose childhood abuse and neglect [4, 8].

In Canada, healthcare professionals are mandated to report suspected CM to child welfare [9]. Healthcare providers are on the frontlines to detect CM and EDs are critical entry points for children who present with injuries due to more severe cases of abuse or neglect [10, 11]. However, the number of child abuse reports from EDs tends to be low [9, 12, 13] and problems with identification methods have been noted [14, 15]. The rates of child abuse and neglect in children presenting to ED have been reported from 0.1–2%, although older studies have reported rates as high as 10% [16]. Further, evidence suggests that 20 to 30% of children who died from CM had previous documented health care visits [17]. In Canada, a limited number of studies have examined children presenting to EDs and these have focused on brain or head injuries related to CM [18–20].

An understudied source of CM surveillance data is the Canadian Hospitals Injury Reporting Prevention Program (CHIRPP). CHIRPP is a sentinel surveillance program that collects and analyzes information on injuries and poisonings of patients who present to the EDs of participating hospitals (currently 19) across Canada. Historically, CHIRPP has not been used as a source of CM surveillance, however, recent efforts have been made to explore intentional injury reports in CHIRPP [21]. Due to its focus on pediatric hospitals, CHIRPP potentially provides a valuable source of CM data. To our knowledge, there are no studies that have exclusively examined child maltreatment using the CHIRPP database. This study: (1) describes CM cases identified in the CHIRPP database between1997/98 to 2010/11; (2) assesses the level of CM case capture over the 14-year period and; (3) uses content analysis to identify additional information captured in text fields.

## Methods

### Sample

The CHIRPP is a sentinel surveillance program (injuries and poisonings) operating in the EDs of 11 pediatric and eight general hospitals participating in CHIRPP across Canada. When an injured person presents to a participating emergency room, they (or the accompanying caregiver) are asked to complete a one-page questionnaire including their accounts of the circumstances surrounding the injury (“what went wrong”) [22, 23]. The attending physician or hospital staff add clinical details such as the injured body part, the nature of the injury (e.g. fracture, concussion, poisoning), whether the injury was intentional (i.e. sexual assault, maltreatment), unintentional (i.e. accidental), or undetermined/unknown, the location where the injury occurred, and any treatment received. These details along with extracts from patients’ accounts are entered into the CHIRPP database by data coders [23]. Patients’ accounts of the injury event are condensed into text fields (maximum 120 characters for the time period of this study).

### Eligibility criteria

In CHRIPP data there are two codes that identify violence against children. Cases associated with CM were selected if: (1) the intent was coded as ‘sexual assault by bodily force’ (CHIRPP intent code 12) or ‘maltreatment by a parent or caregiver’ (CHIRPP intent code 13) or; (2) the text field contained various keywords (e.g. “abuse”, “rape”, “assault”) identifying any cases which may have been misclassified; (3) the patient was under 16 years (191 months) and; (4) the injury occurred between April 1, 1997 and March 31, 2011. This period was chosen for consistency of coding and data capture [22, 23]. The CHIRPP database underwent an upgrade in 1996 and again in 2011 to eCHIRPP (an electronic version of the surveillance system).

### Time trend analysis

To quantify CHIRPPs capture of CM cases over the study period, the data were normalized:

for each year the number of CM cases for children less than 16 years (0–191 months) was divided by the total number of CHIRPP cases for children less than 16 years of age, times 100,000.

Joinpoint regression [24] was used to assess the trend by locating inflection points (joinpoints) and calculating the Annual Percent Change (APC) of each identified segment according to the methods described by the National Cancer Institute [25]. Joinpoint regression software tests whether the APC of each segment is significantly different from zero at the alpha = 0.05 level and produces a 95% confidence interval (CI). The weighted average (AAPC) was also calculated for the entire time span.

### Coding text fields

#### Codebook development

Content analysis [26] was used to examine text fields to identify additional data from CM cases identified through the CHRIPP codes. A codebook was designed by four researchers (AC, EG, LT and MH) to establish consistent guidelines in order to abstract data from text fields. A 10% random sample of text fields in English and French was removed from the overall sample and each coder independently identified key themes, patterns, and emerging trends in order to develop their own version of a codebook. All codebooks were shared among the coders for discussion and feedback. Once core variables were identified, definitions, guidelines and examples were developed. Decisions were then made regarding which codes to keep, expand, or exclude, resulting in a single version of the codebook for reference purposes. For example, the category first identified as “injury due to exposure to IPV” was expanded to “injury due to exposure to family violence” as some cases reported violence between family members other than intimate partners or between family members and other adults. In these cases, the child or adolescent was physically harmed as a result of the violence.

#### Codebook testing

In order to test consistency of code application, two teams of coding partners (AC, EG and LT, MH) independently coded the first 100 text fields and compared results. When it was established that coders were using the codebook consistently, coding of the text fields in the dataset began. Minor changes were made to coding guidelines at this stage as questions arose about specific coding decisions, and to be inclusive of emerging data patterns.

#### Application of codebook

Once testing of the codebook was completed, text fields were divided between two coding teams (1100 for each team) and recorded into separate Excel files. One group of coding partners coded English and French text fields, while the second coded English only. Coding partners compared their coding decisions for all variables and calculated agreement for type of maltreatment. Overall, inter-coder agreement was 85% for type of CM. Although inter-coder agreement was not calculated for all variables, each was checked by coding partners to ensure consistency. When discrepancies occurred, coding partners discussed the issue and made a joint decision as to how to proceed. Any discrepancies or questions that could not be resolved between coding partners were brought to the other team members and discussed until consensus was reached.

Once both coding teams completed their checks, descriptions of coded variables were consolidated into one excel sheet along with existing CHIRPP variables (e.g. age, gender) and assigned numerical values. Written descriptions of certain variables (i.e., additional information) were kept in separate excel files with a corresponding ID number for reference purposes. Descriptive statistics were then conducted to assess the frequency of selected variables identified from text fields. Finally, the frequency of types of CM were presented by age and gender.

## Results

### Eligible cases

A total of 2112 CM cases were identified using intent codes for children under 16 years over a 14-year period (1997/98 to 2010/11). In addition, 96 cases were identified through a keyword search. Six duplicates and two miscoded cases were excluded, resulting in a final sample of 2200 cases representing 11% of intentional injury cases for this period. The majority of the patients in the overall sample were female (59%) and the median age was 7.8 years.

### Time trend analysis

**Fig. 1:**
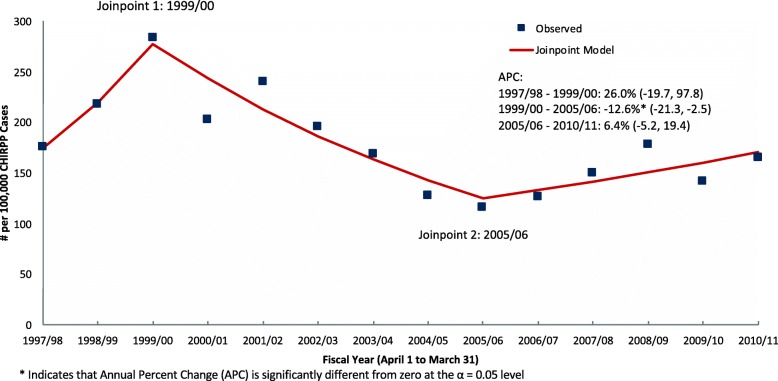
Joinpoint analysis of child maltreatment cases, CHIRPP 1997/98 to 2010/11, ages 0–15 years, both sexes. APC: Annual Percent Change

Figure 1 shows the results of the Joinpoint regression analysis. Two joinpoints were identified: 1999/00 and 2005/06. Between fiscal years 1997/98 and 1999/00 there was a non-significant increasing trend of 26.0% (CI: − 19.7,97.8). Between 1999/00 and 2005/06 there was a decreasing trend of 12.6% (CI: − 21.3, − 2.5). From 2005/06 to 2010/11 there was a slight increase of 6.4% (CI: − 5.2, 19.4), but this was not statistically significant. The overall average annual percent change (AAPC) was not considered to be an appropriate representation of the trend and is not reported.

### Text field themes

Table 1 presents unique information obtained from text fields. Of the sample, 680 (31%) cases were the result of ‘sexual assault by bodily force’ and 1520 (69%) were the result of ‘maltreatment by a parent or caregiver.’ When categorized by CM type, injury due to physical abuse was the most prevalent form of maltreatment, followed by sexual assault, unspecified maltreatment, injury due to exposure to family violence and neglect. Emotional maltreatment was never recorded on its own but identified as the co-occurring form of abuse in less than 1% of cases (data not shown). Co-occurring abuse was identified in 5% of cases (data not shown).

**Table 1:** Distributions of Variables Identified in CHIRPP Text Fields, n (%)

Text Field Variables	Sexual Assault by Bodily Force	Maltreatment by Parent or Caregiver	Total Cases
Total Cases	680 (31)	1520 (69)	2200 (100)
Maltreatment Type			
Sexual Assault	680 (100)	0	680 (31)
Physical Abuse	0	1264 (83)	1264 (57)
Exposure to Family Violence	0	55 (4)	55 (3)
Neglect	0	46 (3)	46 (2)
Unspecified	0	155 (10)	155 (7)
Fatalities	0	8 (0.5)	8 (0.4)
Relationship to Perpetrator			
Family Member			
Parent	−^a^	–	145 (7)
Father or Stepfather	79 (12)	600 (39)	679 (31)
Mother or Stepmother	–	–	404 (18)
Relative (Male)^b^	38 (6)	13 (0.9)	51 (2)
Relative (Female)^c^	–	–	20 (0.9)
Other Family Member^d^	27 (4)	5 (0.3)	32 (1)
Non-family Member in Caregiving Role			
Foster Parent	0	8 (0.5)	8 (0.4)
Partner of Parent (Male)	16 (2)	47 (3)	63 (3)
Teacher or Day-Care Worker	7 (1)	13 (0.9)	20 (0.9)
Babysitter	8 (1)	23 (2)	31 (1)
Known Peer/Adult			
Friend	36 (5)	0	36 (2)
Boyfriend/Ex-boyfriend	9 (1)	0	9 (0.4)
Neighbor	14 (2)	0	14 (0.6)
Acquaintance	17 (3)	0	17 (0.7)
Other Known Peer/Adult	86 (13)	15 (1)	101 (5)
Unknown Peer/Adult			
Stranger	55 (8)	0	55 (3)
Drug/Alcohol Use	98 (14)	43 (3)	141 (6)
Non-Health Care Professional			
Child Welfare	7 (1)	83 (5)	90 (4)
Other Non-Health Care^e^	–	–	29 (1)
Maltreatment During Visitation	23 (3)	64 (4)	87 (4)
Removal of Child	–	–	26 (1)

^a^ Cases less than 5 indicated with a dash due to suppression rules for small cell counts

^b^ Relative (male) includes brothers, grandfathers and uncles

^c^ Relative (female) includes sisters, grandmothers and aunts

^d^ Other family members include cousins & other relatives with unspecified gender

^e^ Non-health care professional includes law enforcement and educational professionals

The CHIRPP disposition code for fatalities, combined with data from text fields, revealed that eight children (< 1%) died from CM. These fatalities occurred mostly among male children ranging in ages from 0 to 3.5 years from head related injuries (data not shown).

Of cases involving ‘sexual assault by bodily force’ the majority of reported perpetrators were known peers or adults followed by fathers or stepfathers and other male relatives. Friends were reported as perpetrators more often than a boyfriend or ex-boyfriend. Strangers were identified as perpetrators in 8% of sexual assault cases.

Of cases involving ‘maltreatment by a parent or caregiver’, family members, specifically fathers or step-fathers, followed by mothers or step-mothers and parents, were reported as responsible for injury due to maltreatment in the majority of cases. The male partner of a parent (i.e. mother’s boyfriend) was identified most often among non-family members in a caregiving role.

Drug and/or alcohol use by perpetrators, victims or both was identified in 6% of all cases; the majority of which were for sexual assaults.

Non-health care professionals were identified as involved in 5% of cases. The majority of these cases indicated the involvement of child welfare (4%) for ‘maltreatment by a parent or caregiver.’ Other non-health care professionals, such as law enforcement, were identified as involved in less than 1% of cases.

In 4% of cases, maltreatment reportedly occurred during a visitation period with a parent or step-parent. Finally, children were reportedly removed from their home in just over 1% of cases.

### Distribution of CM type by age and gender

Table 2 presents the frequencies of CM types by age and gender. Female patients accounted for 88% of sexual assault cases while male patients tended to have slightly higher prevalence of physical abuse, neglect, and injury due to exposure to family violence. Physical abuse and sexual assault were more prevalent among older children (aged 12–15 years), while younger children were more likely to be brought to ED for neglect (1–3 years) and injury due to exposure to family violence (< 1 years). Sexual assaults were identified among all age groups with children under the age of one, followed by children 8–11 years showing the lowest levels.

**Table 2:** Child Characteristics by Primary Maltreatment Type, n (%)

Characteristic	Maltreatment Type					Total
	Sexual Assault	Physical Abuse	Neglect	Exposure to Family Violence	Unspecified	
Total	680 (30.9)	1264 (57.5)	46 (2.1)	55 (2.5)	155 (7.0)	2200 (100)
Gender^a^						
Female	597 (87.8)	590 (46.7)	22 (47.8)	25 (45.5)	69 (44.5)	1303 (59.2)
Male	83 (12.2)	673 (53.2)	24 (52.2)	30 (54.5)	86 (55.5)	896 (40.7)
Age Group						
< 1	7 (1.0)	165 (13.0)	14 (30.4)	22 (40.0)	55 (35.4)	263 (12.0)
1–3	111(16.3)	222 (17.6)	22 (47.8)	14 (25.5)	65 (41.9)	434 (19.7)
4–7	154 (22.7)	278 (22.0)	7 (15.2)	7 (12.7)	21 (13.5)	467 (21.2)
8–11	60 (8.8)	232 (18.4)	−^b^	6 (10.9)	–	309 (14.0)
12–15	348 (51.2)	367 (29.0)	–	6 (10.9)	–	727 (33.0)

^a^ One case did not specify gender

^b^ Cases less than 5 indicated with a dash due to suppression rules for small cell counts

## Discussion

This study is the first to comprehensively assess CM cases in CHIRPP, including text field data. A total of 2200 CM cases were identified for children under 16 years between 1997/98 and 2010/11. Our time trend analysis revealed a significant decrease in the capture of CM cases between 1999 and 2005. Text fields captured unique information such as types of CM resulting from direct physical injury as well as perpetrator characteristics and other details surrounding the injury event.

Joinpoint regression analysis was used to assess the trends in the capture of CM cases over the 14-year period. The main finding was a significant decrease in capture between 1999/00 and 2005/06. Child maltreatment (and other intentional injuries) has traditionally been difficult to capture in CHIRPP since the system was mainly designed to capture unintentional pediatric injuries [21–23]. It is possible that the decrease was partially due to difficulty in capturing these cases in some CHIRPP sites due to administrative and sensitivity issues. Recent improvements in the CHIRPP system [23] and efforts to understand and increase acquisition of intentional injuries [21, 27] will help to improve case capture. The increasing, but not significant, trend in the last segment 2005/06 to 2010/11 suggests that case identification may be increasing, but continued surveillance is needed to analyze the trend beyond the study period.

Physical abuse was the most common type of CM captured in CHIRPP, followed by sexual assault. Our findings regarding the distribution of CM types are somewhat consistent with both Canadian and international ED studies that examine multiple types of CM [28–30]; although differences in timing of studies, sample size, age-ranges, and categories of maltreatment assessed, make direct comparisons challenging. Comparisons with child welfare data are also challenging since they illustrate a different picture of CM in Canada. In our study the prevalence of physical abuse and sexual assault were higher than reported in Canadian child welfare data, while exposure to family violence and neglect were comparatively low [7]. In CHIRPP, emotional maltreatment was rarely identified, and never in isolation, but made up 9% of substantiated child welfare investigations in 2008 [7]. Emotional maltreatment and neglect are difficult to capture in an ED setting [31]. For instance, neglect can present as physical injury from ingestions or inappropriate supervision [16], but intent is challenging to capture and emotional maltreatment rarely manifests in physical injury. Cases of co-occurring CM are well established in the literature, [7, 32–36] but were not well captured in CHIRPP. The low prevalence of co-occurring abuse in CHIRPP may be due to the fact that cases are identified through a specific injury event that requires immediate medical attention, and not through an investigation or assessment of a household.

In this study, a minority of cases indicated involvement with child welfare and 1% of children were removed from their homes. As CHIRPP does not usually provide information as to whether the child is receiving services from child welfare, or information regarding what happen after the initial injury event, our findings are likely underestimates. Our findings are however consistent with studies regarding health care professionals under-reporting CM to child welfare services [9] although an Ontario study found that the proportion of referrals to child welfare agencies for CM investigations from hospitals doubled between 1993 and 2013 [37]. An unexpected finding in our study was the number of cases that indicated injury due to CM during a visitation with a parent or step-parent. Although we did not find studies that dealt with this issue directly, child welfare data indicates that 13% of CM related investigations noted a child custody dispute [38].

Some patterns identified in our study were consistent with previous studies. As supported by the literature from Western countries, girls were significantly more likely to experience sexual assault [8, 28, 32, 39, 40]. In our study, sexual assaults increased for children age 1–7 years and increased again for adolescents 12–15 years. This may demonstrate a pattern whereby family members perpetrating sexual assault are more likely to abuse younger children and non-family members, such as peers, dating or romantic partners, became the predominant perpetrators of sexual assault in adolescents. Abuse perpetrated against older children could also indicate a pattern of re-victimization [41, 42]. Consistent with previous studies our study also found that in cases of sexual assault perpetrators were more likely to be relatives, or known peers or adults, than strangers [43, 44].

Alcohol and drug use were identified in CM cases among both victims and perpetrators, especially in cases involving sexual assault. Child welfare data indicates that alcohol and drug abuse are risk factors for perpetrators in substantiated CM investigations [7, 45]. Previous research has shown a strong relationship between alcohol and sexual assault among female college students, and some evidence indicates that adolescent girls are more likely to experience physical force in alcohol-related sexual assaults than non-alcohol-related assaults [46].

In this study, fatal injuries occurred almost exclusively among young male children (< 4 years) from head related trauma. These findings are consistent with previously published Canadian studies, [18–20] and international studies [5, 28, 30, 47] that examined head injuries highlighting the young age and gender of patients. Traumatic brain injury in particular is associated with high mortality and morbidity in infants. Younger children are also more likely to experience prolonged consequences from their injuries [18, 48]. Also consistent with previous hospital studies [49], we found that fatalities among children in maltreatment categories were higher than other unintentional injury cases in CHIRPP (0.4% vs. 0.1%). The higher prevalence of fatalities among children admitted to the ED due to CM in CHIRPP speaks to its importance as a surveillance tool for this vulnerable population.

### Limitations

There are a number of limitations to this study. CHIRPP is not representative of the Canadian population making it challenging to compare with other CM data. Information provided in text fields are based on patients’ interpretations of the questions and could be written or censored by patient’s caregivers. Further, accounts of the injury event could be potentially flawed from recall errors and omissions. Lack of training of hospital staff and clinicians may impact their ability to recognize all types of CM and can result in under-reporting [50]. In addition, CM that does not result in serious injury is not captured in CHIRPP. It is also possible that less severe cases were misclassified and not identified by our search strategy. Physical assault directed toward children and adolescents from non-family members, or those not in a caregiving role, could be missed due to current CM intent codes. Although fatalities were captured in our study, CHIRPP is generally a poor source of fatalities because of the lack of information of cases past the initial injury event and because some cases bypass the ED altogether due to the severity of injuries.

### Strengths

CHIRPP provides on-going, timely and detailed clinical data on different types of CM. Our study was conducted on a broader age-range of children and adolescents than previous studies. In addition to providing clinical details, CHIRPP text fields offer unique, case specific data which can provide details of the complexities of the injury event, including risk and protective factors [23]. Text fields serve to identify rare events and to increase the granularity of coding.

The examination of both CM codes and the accompanying text fields allows for a more detailed understanding of other CHIRPP categories (e.g. fatalities). At the time that these cases were recorded (1997/98–2010/11), text fields were limited to 120 characters. The electronic application of CHIRPP (eCHIRPP) launched in April of 2011, has an extended text field of 4000 characters providing the opportunity for more detailed information of the injury event. This expanded text field provides further opportunity to present details of complex cases.

### Recommendations

The electronic application of CHIRPP, including its integrated data management tools, has enhanced timeliness and flexibility providing opportunities to enhance CHIRPP data collection [23]. In future, exploring the feasibility of expanding eCHIRPP to include all 5 types of CM intent code classifications would be valuable. Once CM has been identified using eCHIRPP, health care providers could be presented with a “mark all that apply” check list of each type of CM. This would allow for more in-depth capture of different types of CM and identify cases of co-occurring maltreatment. In addition, the term “sexual assault” could be reviewed and redefined as “sexual violence” to better reflect the diversity of cases perpetrated against children and adolescents. The analysis of CM in CHIRPP, however, should also include an analysis of text field data taking advantage of new techniques such as machine learning whenever possible. Our study has shown that they provide valuable details, and context, not available from the CHIRPP survey checklist alone.

## Conclusion

In Canada, national sources of CM data are limited and the ability to examine the magnitude of this problem depends on the availability of diverse sources of data. While further research is required, the findings from this initial study indicate that CHIRPP could be a complimentary source of CM data. Although the examination of text fields increased the capture of different types of CM, CHIRPP is better suited to capture physical abuse and sexual assault. Further, the text field component of CHIRPP provides unique details of the CM injury event, in the majority of cases. This additional information also provides an opportunity to establish potential risk factors and to focus prevention and support to those exposed to CM.

## Data Availability

Data for CHIRPP are publicly available http://www.phac-aspc.gc.ca/injury-bles/chirpp/index-eng.php by contacting the Centre for Surveillance and Applied Research, (Injury.Surveillance@phac-aspc.gc.ca).

## Abbreviations

APC/AAPC: Average Annual Percent Change
CHIRPP: Canadian Hospitals Injury Reporting Prevention Program
CIS: Canadian Incidence Study of Reported Child Abuse and Neglect
CM: Child maltreatment
ED: Emergency department
PHAC: Public Health Agency of Canada
